# HCV and Lymphoproliferation

**DOI:** 10.1155/2012/980942

**Published:** 2012-07-19

**Authors:** Anna Linda Zignego, Carlo Giannini, Laura Gragnani

**Affiliations:** ^1^Center for Systemic Manifestations of Hepatitis Viruses (MASVE), Department of Internal Medicine, University of Florence, 50134 Florence, Italy; ^2^Istituto Toscano Tumori (ITT), 50139 Firenze, Italy

## Abstract

Hepatitis C virus (HCV) infection is a serious public health problem because of its worldwide diffusion and sequelae. It is not only a hepatotropic but also a lymphotropic agent and is responsible not only for liver injury—potentially evolving to cirrhosis and hepatocellular carcinoma—but also for a series of sometimes severely disabling extrahepatic diseases and, in particular, B-cell lymphoproliferative disorders. These latter range from benign, but prelymphomatous conditions, like mixed cryoglobulinemia, to frank lymphomas. Analogously with *Helicobacter pylori* related lymphomagenesis, the study of the effects of viral eradication confirmed the etiopathogenetic role of HCV and showed it is an ideal model for better understanding of the molecular mechanisms involved. Concerning these latter, several hypotheses have been proposed over the past two decades which are not mutually exclusive. These hypotheses have variously emphasized the important role played by sustained stimulation of the immune system by HCV, infection of the lymphatic cells, viral proteins, chromosomal aberrations, cytokines, or microRNA molecules. In this paper we describe the main hypotheses that have been proposed with the corresponding principal supporting data.

## 1. Introduction

Hepatitis C virus (HCV) infection is a major public health problem with an estimated 3-4 million people infected each year worldwide and about 170–200 million carriers. These latter are at risk of developing liver cirrhosis and/or liver cancer. More than 350,000 people die from HCV-related liver diseases each year. Moreover, these estimates do not take into account the extrahepatic aspects of HCV infection.

 Early after its discovery, it was shown that HCV is also a lymphotropic virus [1]. As a consequence of the lymphatic infection, several lymphoproliferative disorders (LPDs) have been associated with this virus [2], including mixed cryoglobulinemia (MC), B-cell non-Hodgkin's lymphoma (NHL) [3–10] and monoclonal gammopathies [11–13].

Mixed cryoglobulinemia is the most frequent and well known LPD developing during HCV infection. Although clinically benign, MC is a prelymphomatous disorder leading to NHL in about 5–10% of cases. This makes MC a valuable model for study of pathogenetic mechanisms of HCV-related LPDs [2–14]. MC was previously interpreted as a lymphoma in situ, being characterized by bone marrow and/or liver infiltrates closely resembling NHL [15]. Therefore, it was hypothesized that HCV may be involved in the pathogenesis of NHL as well [1, 4]. This hypothesis was substantiated by several observations, including the significantly high prevalence of HCV infection in NHL patients in several studies [6, 7, 10, 12, 16–18]. A lot of data are presently available showing, in most cases, a significant association with B-cell NHL, even with a clear south-north gradient and involving different histopathological types of lymphoma, the most strictly associated being the lymphoplasmacytic, marginal zone and diffuse large B-cell lymphoma [19]. A case-control study has shown that HCV infection increases the risk for NHL involving the liver and major salivary glands by about 50-fold (i.e., a risk higher than that for hepatocellular carcinoma) and the risk for NHLs at other sites by about 4-fold [20].

The observation of the effect of viral eradication using antiviral agents strongly supports the etiopathogenetic link between HCV infection and lymphomagenesis. Analogously with what has been reported for MC, in the case of low grade B-cell lymphoma—and especially in cases of splenic lymphoma—clinical remission following effective antiviral therapy in HCV-associated cases has been observed [21–23].

Interestingly, in a recent Japanese study involving about 3,000 HCV-infected patients observed during a long-term follow-up, it was shown that the annual incidence of lymphoma was 0.23% and the cumulative rate of lymphoma development after 15 years was 2.6% in both the untreated and non-responder patients with persisting infection versus 0% in treated patients achieving viral eradication, strongly suggesting that antiviral therapy protects against the development of lymphoma [24].

## 2. Mechanisms of HCV-Related Lymphomagenesis

**Figure 1:**
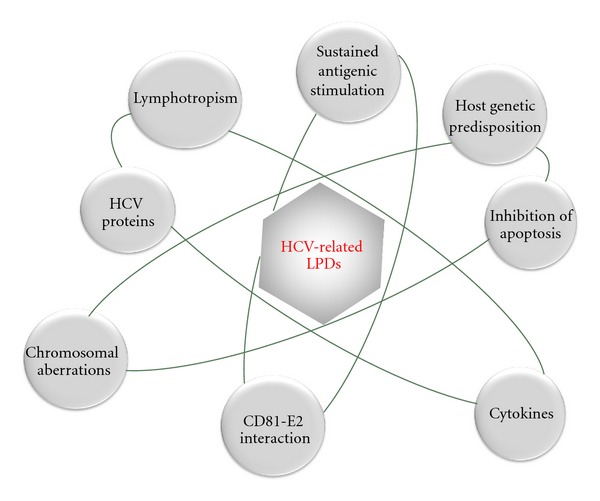
Pathogenesis of HCV-related lymphoproliferative disorders (LPDs). Main working hypotheses and their principal interconnections.

Several hypotheses, frequently interconnected with each other, have been proposed in regard to the possible mechanisms of HCV-related lymphomagenesis (Figure 1). These include a key role played by the sustained antigenic stimulation of the B-cell compartment, the role of viral lymphotropism and viral proteins, chromosomal aberrations, cytokines, and microRNAs.

### 2.1. The Role of Sustained Antigenic Stimulation

**Figure 2:**
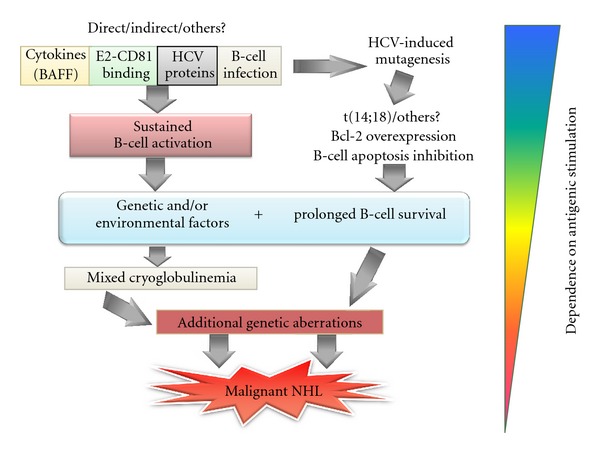
HCV-related LPD pathogenesis is a multifactorial and multistep process. Current data suggest that the starting points of this process are represented by the cooperation between a sustained and persistent stimulation—by direct or indirect action of viral particles or proteins—and antiapoptotic mechanisms acting on B-cell compartment. A predisposing genetic background would be responsible for the final evolution to a particular LPD (namely, MC). The progressive addition of genetic aberrations would lead to a frank neoplastic transformation, gradually making the process less dependent on the etiologic agent.

Sustained HCV-driven antigenic stimulation has been suggested to play a key role in inducing B-cell clonal expansion characterizing these disorders (Figure 2). The presence in the liver of lymphatic structures resembling lymphatic follicles is characteristic of HCV infection. It has been suggested that they represent an important site of B-cell clonal expansion, especially in patients with MC, where they have been found in almost all cases [25]. Furthermore, B lymphocytes isolated from hepatic follicles produced rheumatoid factor (RF) that most frequently display the WA cross-reactive idiotype, considered to be characteristic of MC [25]. In particular, it was observed that intrahepatic B-cell clonalities were invariably associated with extrahepatic manifestations of HCV infection, including high serum levels of RF activity, cryoglobulins, monoclonal gammopathy of undetermined significance (MGUS), and frank B-cell NHL [26]. The key role of antigen-driven stimulation in HCV-related lymphoproliferation was also supported by a study investigating mutations in the V(H) and V(K) genes of the B-cell clone inducing a frank NHL in an MC patient and producing an IgM homologous to a protein with RF specificity. The observation of an IgH ongoing mutation process and the expression of an Ig antigen receptor significantly homologous to an anti-HCV protein suggested that both MC and NHL were antigen-driven LPDs sustained by HCV [27]. The analysis of bone marrow specimens from the same patient taken at different times during the evolution from MC to NHL showed a marked reduction in intraclonal diversity at the stage of overt NHL, indicating a minor dependence of the cells on the antigen-driven mechanism. Such a progressive independence from the initial etiologic agent may be deduced also by the effects of viral eradication in patients with variable severity of the HCV-related LPD (see also the following). Analogously with *Helicobacter pylori*-related lymphomagenesis, it is conceivable that, during the multistep lymphomagenic process, progressive independence from the antigen-driven mechanism will develop, possibly due to the occurrence of chromosomal translocations or other genetic aberrations [14, 28] (Figure 2).

It has also been suggested that the same HCV antigens may be involved in the induction of both MC and lymphoma [27, 29]. The viral antigen/s responsible for B-cell clonal expansion are not perfectly defined yet. However, De Re and colleagues showed that, in patients with MC and immunocytoma, the B-cell receptor (BCR) of the monoclonal, overexpanded B-cell population, as well as the IgM-RF+ component of the cryoprecipitate, showed cross-reactivity against HCV NS3 antigen [30].

Other studies have focused on the possible role of proteins of the HCV envelope and mainly on HCV E2 protein (Figure 2). It has been shown that E2 interacts with the tetraspanin CD81, present also on the B-cell surface. This binding has been suggested to be responsible for sustained polyclonal B-cell activation essentially by lowering the B-cell activation threshold [31, 32].

Data supporting the hypothesis that some HCV-associated lymphomas could originate from B cells that were initially activated by the HCV-E2 protein have been provided. In fact, Quinn and coworkers showed that the immunoglobulin from one of two HCV-associated lymphomas they tested bound the E2 protein in a manner identical to a *bona fide* human anti-E2 antibody, hypothesizing that the B-cell activation derived from the dual binding of E2 to a cognate BCR and to the CD81 molecule, which is a component of a signaling complex [33].

Interestingly, the HCV-E2 protein appears to mimic human Ig. In fact, it was observed that the N-terminal region of E2 is antigenically and structurally similar to human Ig variable domains and could represent a target for anti-human IgG antibodies [34]. Consistently, the analysis of the CDR3 sequences of the IgM-RF+ purified from the cryoprecipitate in MC patients allowed the identification of HCV-E2 as the antigen driving the production of IgM-RF [35].

### 2.2. The Role of Viral Lymphotropism and Viral Proteins

The potential role of viral lymphotropism in the pathogenesis of HCV-related LPDs has been emphasized since the first evidence of the presence of viral replication in lymphatic cells [1] (Figure 2). The association between viral infection of peripheral blood mononuclear cells (PBMCs) and the presence of LPDs was initially shown in patients with MC, where more evident infection of PBMCs in comparison with HCV-positive patients without MC was observed [4]. HCV infection was then observed by Galli et al. in bone marrow cells from all patients with MC versus 43% of patients without MC [36]. After these pioneering reports, a large amount of data correlating the presence of HCV in the lymphatic compartment and the development of autoimmune/lymphoproliferative disorders has been produced. In a study using the model of injection of lymphoid cells from HCV-positive patients into SCID mice, it was shown that the samples derived from HCV patients with malignant LPD were characterized by positivity for HCV replicative intermediates, stronger signals when tested for HCV genomic sequences, and successful serial passage of infected cells in different animals [37]. In addition, Sung and coworkers showed the establishment of B-cell lines persistently producing infectious virus from an HCV-positive lymphoma [38].

One interesting point about the actual dimension of lymphocyte infection in HCV+ subjects has been provided by Pal and coworkers who showed HCV infection in 85% of lymph node specimens tested by in situ hybridization and HCV replication in 50% of cases by detection of HCV replicative intermediate [39]. Interestingly, quasispecies analysis in one case indicated that 68% of variants circulating in serum were also present in lymphoid tissues, and only 40% of serum variants were identified in liver, documenting a major contribution of lymphoid replication to HCV viremia [39].

The existence of lymphotropic viral quasispecies has also been demonstrated by elegant studies evaluating HCV IRES sequence in liver and PBMC and its ability to drive viral genome translation in different cell types [40, 41].

The M. Lai's group, using a model of in vitro HCV infection of B-cells, showed that the viral infection may induce an enhanced mutation rate of immunoglobulin genes and some oncogenes, possibly through induction of error-prone DNA polymerase and activation-induced cytidine deaminase (AID), suggesting that HCV may cause tumors by a hit-and-run mechanism [42]. More recently, Ito and coworkers observed a dramatically increased expression of AID in the B-cells of HCV patients, suggesting that this may be a key factor in the lymphomagenetic process mediated by HCV [43]. Furthermore, a significantly higher expression of several lymphomagenesis-related genes in the CD19+ of HCV patients than in controls was also shown [43].

 In regard to viral proteins, particular attention has been focused on the HCV core protein due to previously shown pleiotropic effects on different cell signaling pathways modulating cell viability and proliferation [44]. Focusing on animal models, core transgenic mice developed lymphoma with a high frequency (80%) at ages over 20 months [45]. The core mRNA was shown in the enlarged lymph nodes of the transgenic mice which developed lymphoma. In another transgenic model, where the IFN signaling was disrupted, the inducible and persistent expression of the HCV core in the context of all structural proteins was associated with the development of lymphoid disorders including frank lymphoma, suggesting a synergistic action of the viral proteins with IFN signaling impairment in promoting lymphomagenesis [46]. 

More recently, the expression of the whole HCV genome, restricted to the B-cell compartment, resulted in a high prevalence of diffuse large B-cell lymphoma (DLBCL)(up to 29%) within 600 days after birth [47]. Interestingly, the HCV core gene was expressed in all lymphomas. A wide analysis of the cytokine and chemokine pattern showed elevated levels of serum IL-2R*α* in mice with lymphoma, directly originating from lymphoma tissue [47].

The expression of the HCV core in primary B cells by an adenoviral vector significantly inhibited B-lymphocyte apoptosis and induced a dramatic down regulation of MHC class II molecules. Moreover, genes associated with leukemia and B-lymphoma were consistently up regulated by the HCV core in this model [48].

Finally, in a study performed on both B-cell lines expressing the HCV core protein and in primary B-cells from patients with LPDs, it was possible to show the altered expression of some isoforms of genes of the p53 family, the DNp63 and DNp73, previously shown to be overexpressed in human cancers, including lymphoma [49, 50].

Although ectopic protein expression in both in vivo and in vitro models does not perfectly reproduce the actual situation during chronic viral infection, the consistency and coherence of the data accumulated until now suggest a potential lymphomagenic effect of the core protein when expressed as a single protein or in the context of other viral proteins.

### 2.3. Chromosomal Aberrations

Interesting data are available about the role played by chromosomal aberrations in HCV-related LPDs. The most investigated genetic aberration was the (14;18) translocation—t(14;18)—that was found to be significantly associated with type II or monoclonal MC. The presence of t(14;18) in MC was correlated with the overexpression of the antiapoptotic *bcl-2 *gene in B-cells, resulting in an imbalance of the Bcl-2/Bax ratio and abnormal B-cell survival [51, 52] (Figure 2). The regression of the expanded B-cell clones following effective antiviral treatment and, in some relapsing patients, a new expansion of the same clones were also shown [53]. Furthermore, a long-term follow-up study allowed the identification of occult HCV persistence limited to the lymphatic compartment in some patients resulting sustained viral responders after antiviral therapy [54, 55]. More interestingly, such a persistent occult lymphatic infection was associated with the initial diagnosis of MC, the persistence of some MC symptoms after therapy, and the persistence of expanded t(14;18)+ B-cell clones [54, 55]. The observation of the possibility, even if rare, of a persisting MC disease in spite of complete viral eradication suggested the existence of points of no return in the evolution of the HCV-related lymphoproliferation.

Recently, Goldberg-Bittman et al. reported an increased rate of aneuploidy in chronically infected HCV subjects versus healthy controls, with values similar to an NHL group. This observation suggests that HCV patients could be more prone to develop a lymphatic malignancy also because of bearing such alterations of the ploidy grade [56]. An important contribution to understanding the set of chromosome instability associated with HCV infection was provided by a study of Machida and coworkers. The authors observed a reduced expression of Rb protein—responsible for cell cycle arrest in case of DNA abnormalities—in various conditions including HCV-infected PBMCs isolated from patients, hepatocyte models infected in vitro with HCV or transfected with the viral core protein alone, and transgenic mice expressing core protein. In fact, lower levels of Rb could easily lead to skipping the mitosis checkpoints and contribute to generation of polyploid cells, a condition favoring neoplastic transformation [57]. 

### 2.4. Cytokines, Chemokines, and HCV-Related LPDs

Cytokines and chemokines are essential mediators of the immune response. A disturbance of the equilibrium between activating and repressing effects of these soluble molecules may be responsible for several autoimmune/lymphoproliferative disorders. Numerous reports have suggested that cytokines and chemokines are key factors in the pathogenesis of HCV-related LPDs. The MC model, as prelymphomatous condition, has been widely used to investigate the cytokine pattern characteristic of HCV-related LPDs. The role of Th1 cytokine profile (IFN*γ* and TNF*α*) and some chemokines (MIP-1*α*, MIP-1*β*, CXCL10, and CXCR3) in the pathogenesis of HCV-MC has been suggested by the elevated expression of these mediators observed in vasculitic lesions of MC patients [58]. CXCL13 was another chemokine reported as upregulated in MC patients [59]. This chemokine, also known as BCA-1 (B-cell-attracting chemokine-1) or BLC (B-lymphocyte chemoattractant), is a major regulator of B-cell trafficking, and its expression has been found to be significantly enhanced in microdissected samples from liver biopsy of patients with active cutaneous vasculitis. Antonelli and coworkers showed that serum concentrations of different cytokines and chemokines are significantly modified in MC patients [60] and have recently investigated the potential role of CXCL-10 and CXCL-11 in the pathogenesis of MC [61, 62]. In fact, high serum concentration of these soluble factors has been shown in HCV patients with MC when compared to patients without MC and healthy controls.

A growing body of evidence has accumulated in recent years showing the involvement of the B-cell-activating factor (BAFF or BLyS) in the pathogenesis of HCV-related LPDs (Figure 2). This B-cell-specific cytokine, belonging to the TNF-*α* family, is essential for B-lymphocyte development and survival. Several reports have shown a higher BAFF serum concentration in HCV patients than in healthy controls and, more significantly, in HCV patients with LPDs (for review, see [63]). The mechanisms of the enhanced serum concentration of BAFF in HCV-LPD patients have not been elucidated yet. A possible explanation has been recently suggested by the analysis of the polymorphic variants of the BAFF gene promoter. A particular allelic variant (−871 T), reported to induce an increased transcriptional activity of the BAFF gene [64], was significantly more frequent in patients with HCV-related MC than in HCV patients without MC. The genetic data were corroborated by the presence of higher levels of the cytokine in the serum of MC patients [65, 66].

Concerning HCV-positive subjects with NHL, Libra et al. showed an increase in the circulating levels of IL-1*β* [67]. As well, high serum osteopontin (OPN) levels were associated with B-cell NHL and HCV infection. Interestingly, the highest serum OPN concentrations were found among HCV-infected patients with concomitant type II MC with and without B-cell NHL [68].

### 2.5. MicroRNAs and HCV-Related LPDs

Increasing evidence supporting the role of microRNA (miRNA) deregulation in the pathogenesis of chronic hepatitis C infection and related disorders has been provided in the last years (for review: [69]). MiRNAs are small RNA molecules (19–22-mer noncoding RNAs) able to induce translational inhibition of target genes by partial base complementarity to the target mRNA 3′UTR. A liver-specific miRNA, miR-122, has been shown to be important for HCV replication [70]. It has been shown that interferon treatment can regulate miR-122 and other miRNA levels and that these latter mediate—at least in part—the antiviral effects of IFN [71]. These in vitro data have been partially corroborated by the observation of modified levels of miR-122 in patients unresponsive to the IFN therapy [72]. A few data are still now available concerning the involvement of miRNAs in the pathogenesis of HCV extrahepatic disorders. An interesting report recently analyzed the global miRNA expression profile in HCV-related and unrelated splenic marginal zone lymphomas (SMZLs). By using large-scale miRNA expression profiling analysis, Peveling-Oberhag and coworkers [73] quantitatively evaluated the expression of 381 miRNAs in microdissected SMZLs (HCV-positive and -negative) and in normal splenic tissues. The difference in expression profiles arose between SMZLs and normal spleens for 12 miRNAs, most of which were previously involved in various mechanisms of tumor formation for both lymphomas and other tumors. Only one miRNA, miR-26b, known to show tumor-suppressive activity, was significantly down regulated in HCV-positive lymphomas compared to HCV-negative SMZLs, suggesting a possible specific mechanism by which the virus might unfold its oncogenic potential in malignant lymphoma.

## 3. Conclusions

In conclusion, as summarized in Figure 2, current data suggest that HCV lymphomagenesis is a complex, multistep, multifactorial process, probably based on sustained B-cell-activation and the inhibition of B-cell apoptosis within a background of predisposing genetic factors and evolving through the progressive addition of genetic aberrations which allow the process to become progressively less dependent on the etiologic agent.
